# Methodological Contributions to Educational Research on the Formation of Social Thought

**DOI:** 10.3390/ejihpe12030025

**Published:** 2022-03-16

**Authors:** Delfín Ortega-Sánchez, Carlos Pérez-González

**Affiliations:** 1 Department of Specific Didactics, Faculty of Education, University of Burgos, 09001 Burgos, Spain; 2 Department of Philology, Faculty of Humanities and Communication, University of Burgos, 09001 Burgos, Spain; cperez@ubu.es

The main purpose of social science education is the formation of social thought for the understanding of reality from its critical and creative dimensions and for the intervention and democratic participation of a responsible and committed citizenship; that is, the formation of citizens capable of living democratically with each other and of participating in the social, labor, cultural, and political life of their world, and trying to improve it [1].

The purpose of forming social thought implies overcoming the complexity of social reality through the decoding of the various languages present in society. The critical evaluation of facts, situations, individual, and/or social attitudes—critical thinking—and the proposal of alternatives and solutions to social problems—creative thinking—imply the justification, contrast, and argumentation of opinions and positions so as to intervene socially and to improve individually and collectively [2].

Likewise, the development of social thinking skills takes place in necessarily technological environments, which should be observed, analyzed, and evaluated from an eminently critical perspective. In this sense, the acquisition of skills for the identification/discrimination, evaluation, and appropriate use of information is unavoidable. Achieving this type of thinking implies the educational promotion of digital competencies and equitable technological access. Gender inequality of access is known as the “second digital gap” and is characterized by the low participation of women in the design and programming of software and hardware, as well as by an unequal perception, according to gender, of technological resources [3].

In this context, the present Special Issue answers questions about methodological approaches aimed at addressing social thinking as a research problem in education. It aims to address the following research questions:

1. What type of methodology (qualitative, quantitative, or mixed) would be useful to address the formation of social thought as a research problem in social science education? With what objectives, hypotheses, assumptions, and study variables? In what educational scenarios?
2. How should educational research on the formation of social thought be designed? With what techniques? With what instruments?
3. How are the information society and knowledge society related to the formation of social thinking? What technological resources accompany teachers in the critical education of citizenship? What use of technologies is implemented in the classroom for the achievement of critical thinking? What teaching and learning models, and what uses of educational technology, are reproduced in the classroom to eradicate the gender digital gap?

This research topic includes a small but select article collection on educational research and innovation based on the application of rigorous quantitative, qualitative, and mixed methodological designs, whose results represent a significant contribution to the purpose of this Special Issue.

The importance of the concepts of the information society and the knowledge society in the field of innovation and technology has led to a significant rethinking of the processes of production and the organization of work and the media, in the way we make policy and identify a country’s economic and social development, and in the way we relate to one another. In this area, UNESCO has presented itself as a promoter of the social dimension of technologies, recognizing them as key elements, and supporting the development of national policies and general plans on the use of information and knowledge technologies in education. Its purpose, aimed at helping governments to harness the potential of technologies in education systems, continues to seek to achieve, in particular, Sustainable Development Goal 4—Quality Education, Sustainable Development Goal 5—Gender Equality, and Sustainable Development Goal 10—Reducing Inequalities, all of which are part of the 2030 Agenda. It is, at this point, where the relevance of ‘applied didactic-disciplinary technological literacy’ in teacher training plans, and its specific contribution to the eradication of gender, generational, and social gaps, should be emphasized.

In this context, the first study [4] starts from the need for further development of critical thinking skills in education. The automatism in information access and the assertive dissemination of information, built on biases, stereotypes, and prejudices, motivate the design of training programs aimed at the activation of innovative actions in inclusive education. According to the results obtained, these actions should contemplate the use of digital technologies and their implementation for the acquisition of competencies in critical and social thinking in university training plans, contributing to the eradication of the gender digital divide.

Furthermore, the research by Gómez-Trigueros and Yáñez de Aldecoa [5] analyzes the digital teaching competence of both trainee and in-service teachers, with the purpose of reviewing the teaching methodologies and technological resources used from a gender perspective. With an impressive sample of 914 trainee teachers and 194 university teachers, the results reveal a female continuity in poor differential self-conceptions about their own teaching digital competence and predisposition towards technologies. This study concludes that we must delve into the transformative potential of initial teacher training plans for inclusive comprehensive technological literacy and, in this way, address this challenge from internationally recognized teaching and learning models, such as TPACK.

Finally, also in the field of educational contributions to the Sustainable Development Goals, the research by Díez, Domínguez, Ponsoda, and Ortuño [6] focuses on the analysis of a coherent conceptual intersection between education for sustainable development, education for global citizenship, feminist studies, and digital literacy to work on critical thinking skills and coeducation in future social science teachers. This study, in line with previous studies, demonstrates the usefulness and effectiveness of teacher training programs aimed at educating in and for equality from the earliest educational levels.

The socio-educational evolution towards overcoming the sex–gender system, teacher training in social thought and gender equality, sexual diversity, and coeducation is an essential axis of social action to identify inequality and transform educational practice. From this perspective, it is necessary to agree on strategies and develop preventive campaigns to overcome of differential social discourses based on gender and sexual diversity in face-to-face and/or digital (closed or open) educational environments of social communication.

The contributions to this Special Issue have demonstrated the possibilities of concrete applications of specific concepts, methods, and strategies for educating in and for gender equality to further the development of social thought. In this sense, they support the need to prepare future teachers in gender, digital, and critical competencies for a naturally diverse society. These contributions are added to the international seminars held during 2020 (I—International Seminar on Education and LKT: Interdisciplinary Approaches to Teaching Innovation) and 2021 (II—International Seminar on Coeducation, Gender Digital Gap, and Virtuality in Initial Teacher Training, and III—International Seminar on Education and LKT: Digital Competence, Gender Digital Gap and Virtuality in Initial Teacher Training), organised by more than 15 Spanish national and international European and American institutions. Likewise, these articles are aligned with highly referenced publications in this field. In short, the range of strategies and tools offered by this monograph provide ways to educate students in the means of democratic participation, intervention in social problems, and the implementation of information and communication technologies and learning and knowledge technologies as means and resources that will contribute to this educational purpose.
